# Supplementary material for: Shigella flexneri undergoes niche-specific adaptation during infection
Source: bioRxiv. 2026 Sep 17:2026.09.15.751907. Preprint. [Version 1] doi: 10.64898/2026.09.15.751907 (PMC13596363; doi:10.64898/2026.09.15.751907)
Supplement: Supplementary file 1 [file NIHPP2026.09.15.751907v1-supplement-1.pdf]

bioRxiv preprint doi: https://doi.org/10.64898/2026.09.15.751907; this version posted September 17, 2026. The copyright holder for this preprint (which was not certified by peer review) is the author/funder, who has granted bioRxiv a license to display the preprint in perpetuity. It is made available under a CC-BY 4.0 International license.

Supplementary Information

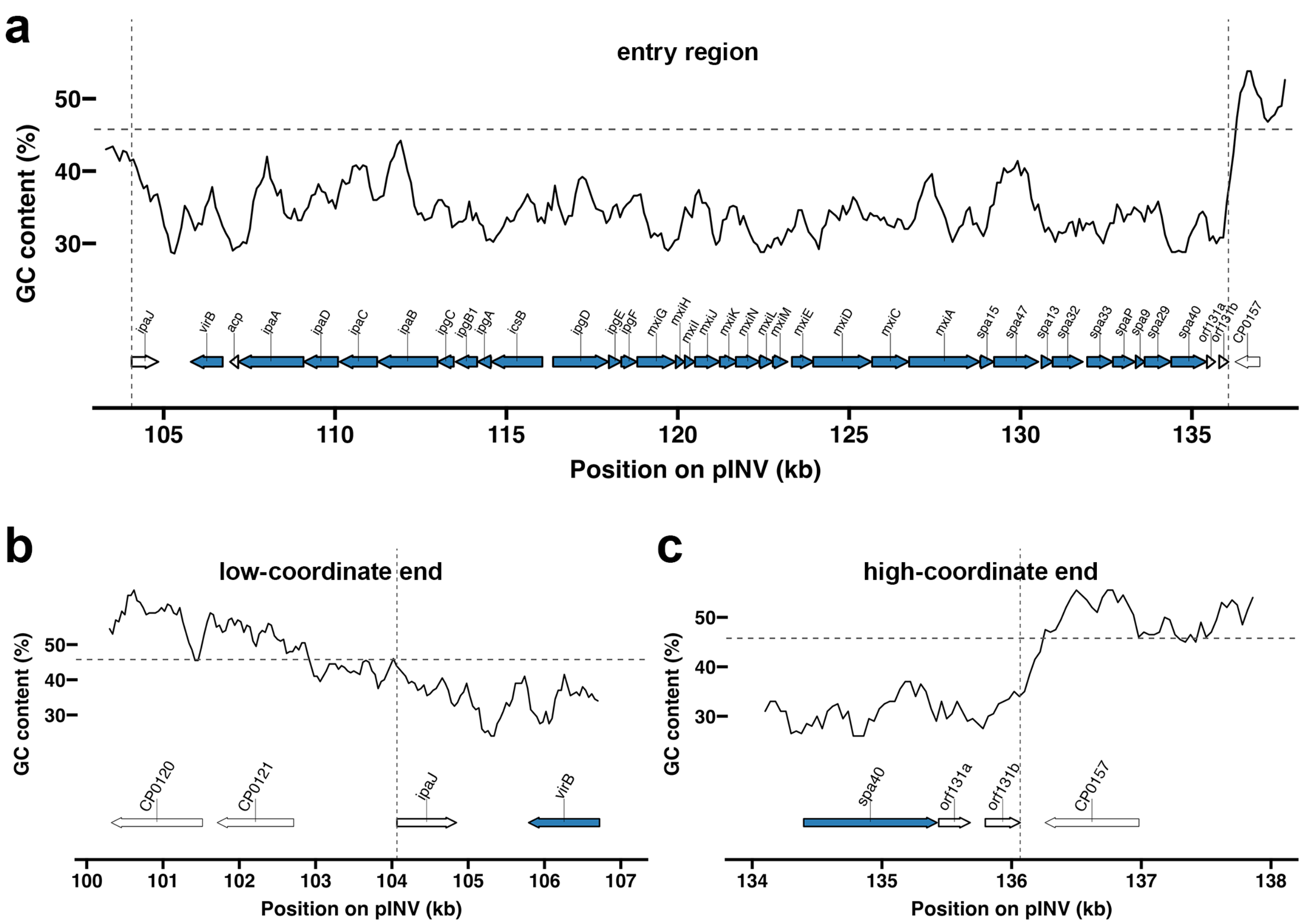

Supplementary Fig 1. GC content across the pINV entry region and its flanking sequences.

GC content is plotted against pINV coordinates using sliding-window analysis, with gene organization shown beneath each plot. Gene arrows corresponding to loci within the pINV entry region have bold outlines, and genes identified as hits in the Tn-seq screen are shown in blue. Vertical dashed lines mark the boundaries of the pINV entry region, and the horizontal dashed line indicates the mean GC content of the entire pINV plasmid. **a** GC content across the pINV entry region and adjacent flanking sequences, calculated using a 500-bp window and a 100-bp step size. **b** GC content across the low-coordinate end of the pINV entry region and its adjacent flanking sequence, calculated using a 200-bp window and a 40-bp step size. **c** GC content across the high-coordinate end of the pINV entry region and its adjacent flanking sequence, calculated using a 200-bp window and a 40-bp step size.

bioRxiv preprint doi: https://doi.org/10.64898/2026.09.15.751907; this version posted September 17, 2026. The copyright holder for this preprint (which was not certified by peer review) is the author/funder, who has granted bioRxiv a license to display the preprint in perpetuity. It is made available under a CC-BY 4.0 International license.

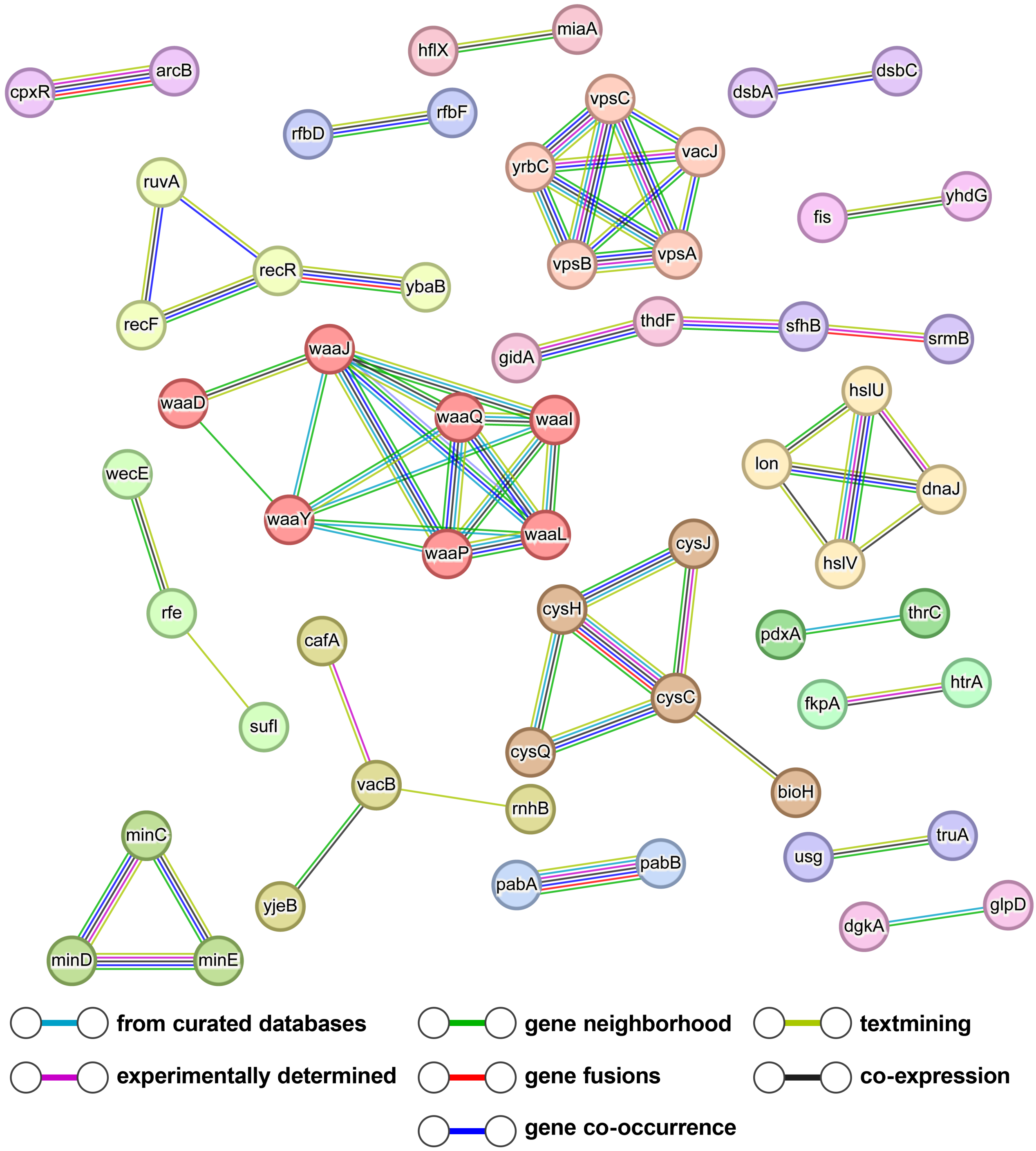

Supplementary Fig. 2: Functional association network of chromosomal Tn-seq hits.

Chromosome-encoded Tn-seq hits were analyzed using STRING with a minimum required interaction score of 0.7. Nodes represent proteins and are colored according to Markov cluster algorithm clusters generated using an inflation parameter of 3. Edges represent known or predicted functional associations, with line colors indicating the supporting evidence sources shown in the key. Disconnected nodes are not shown.

bioRxiv preprint doi: https://doi.org/10.64898/2026.09.15.751907; this version posted September 17, 2026. The copyright holder for this preprint (which was not certified by peer review) is the author/funder, who has granted bioRxiv a license to display the preprint in perpetuity. It is made available under a CC-BY 4.0 International license.

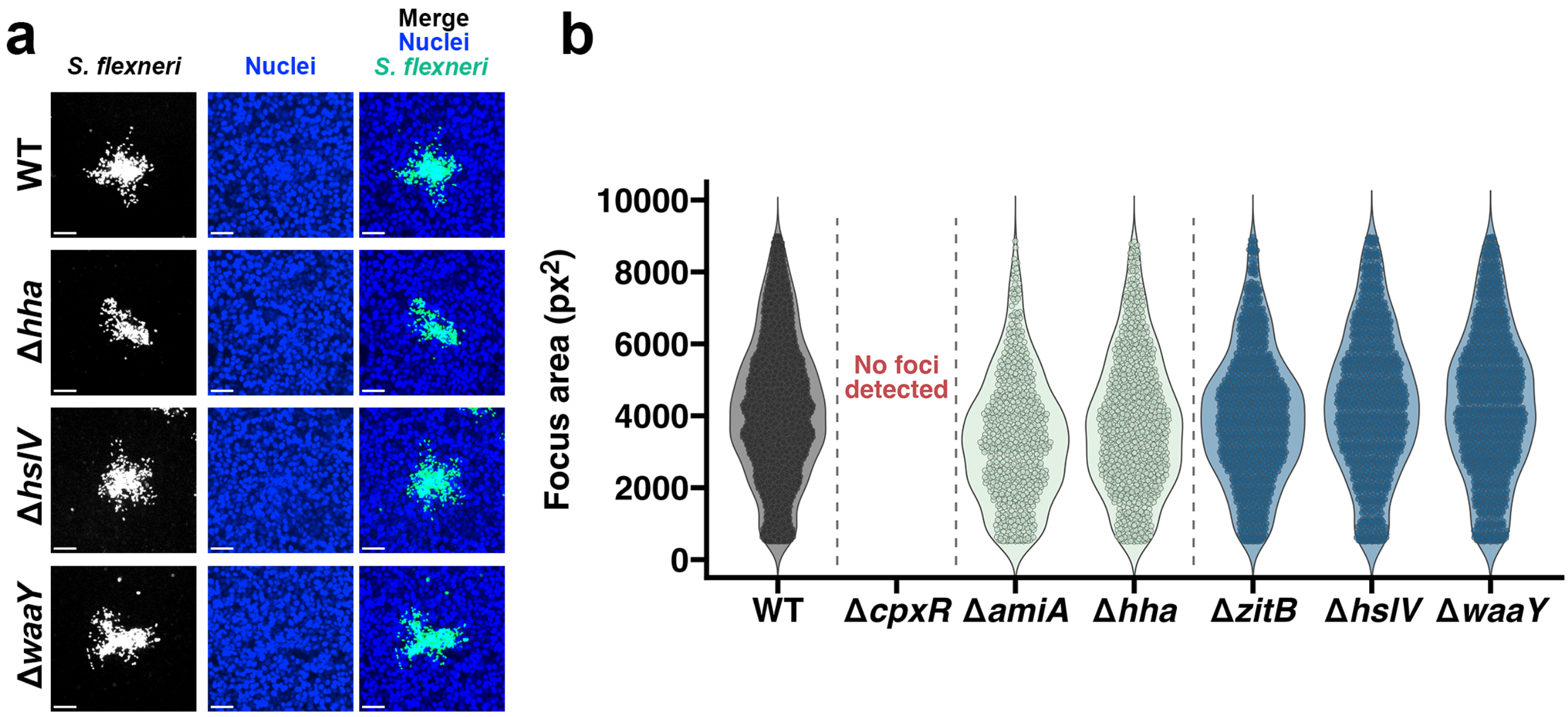

Supplementary Fig. 3: Additional cell-to-cell spread analysis of selected Tn-seq candidate mutants in HT-29 cells.

a Representative images of infection foci formed by WT, Δ*hha*, Δ*hslV*, and Δ*waaY* in HT-29 cell monolayers at 8 hpi. *S. flexneri* is shown in white in the single-channel images and in green in the merged images; nuclei are shown in blue. Scale bars, 50 μm. **b** Violin plots showing the distributions of individual focus areas formed by WT and the indicated mutants in HT-29 cell monolayers at 8 hpi. Each point represents an individual infection focus pooled across three independent experiments (n = 3). No foci were detected for Δ*cpxR*.

bioRxiv preprint doi: https://doi.org/10.64898/2026.09.15.751907; this version posted September 17, 2026. The copyright holder for this preprint (which was not certified by peer review) is the author/funder, who has granted bioRxiv a license to display the preprint in perpetuity. It is made available under a CC-BY 4.0 International license.

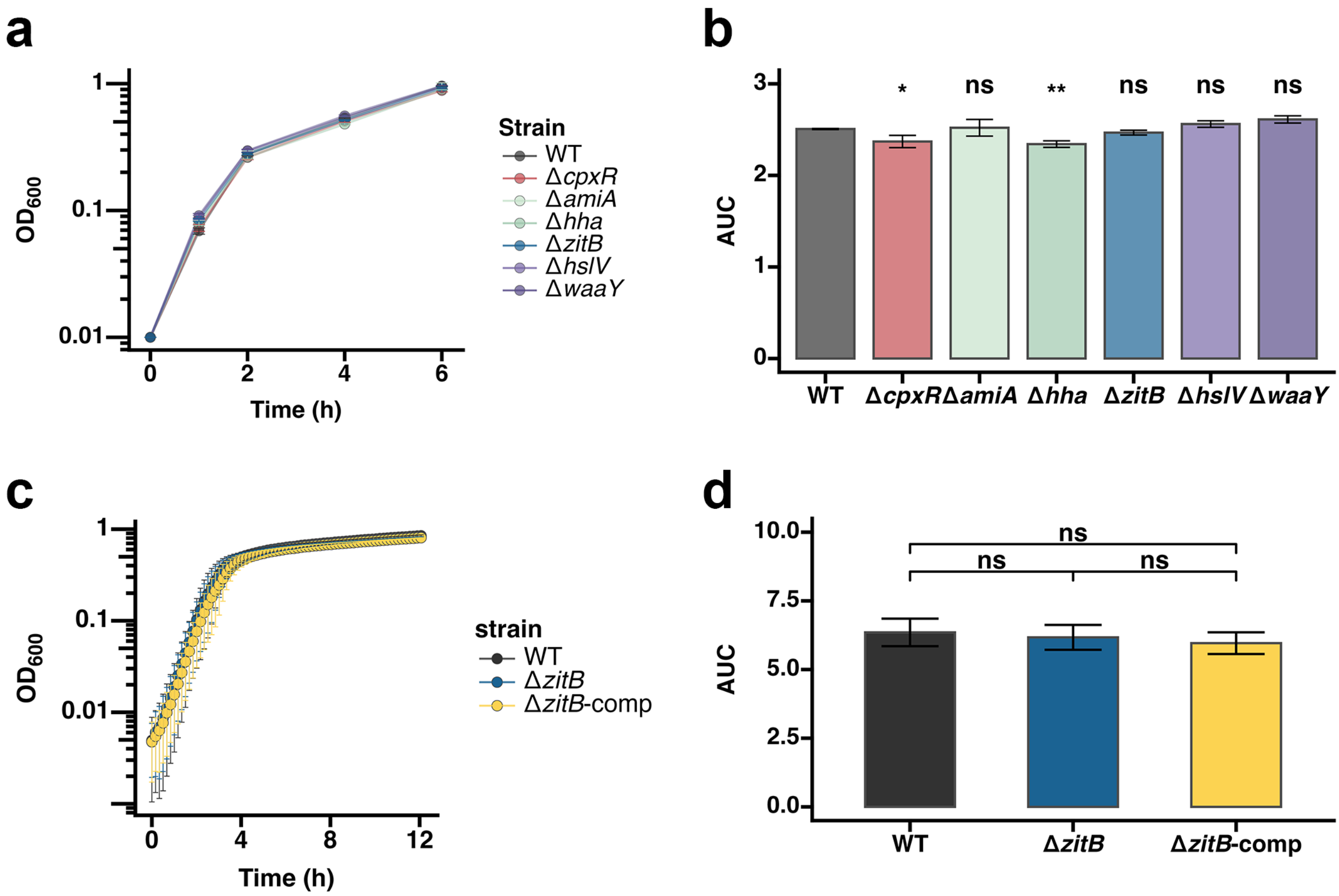

Supplementary Fig. 4: *In vitro* growth of selected strains.

a Growth of WT and the indicated mutants in LB broth, measured by OD_{600} over 6 h. Data are shown as mean ± SD from three independent cultures (*n* = 3). **b** Area under the curve (AUC) calculated from the OD_{600} measurements in **a**. Each point represents one independent culture, and bars show mean ± SD (*n* = 3). Statistical analysis was performed using a linear model followed by Dunnett-adjusted comparisons of each mutant with WT. **c** Growth of WT, Δ*zitB*, and Δ*zitB*-comp in TSB, measured by OD_{600} over 12 h. Data are shown as mean ± SD from three independent experiments (*n* = 3). **d** AUC calculated from the OD_{600} measurements in **c**. Each point represents one independent experiment, and bars show mean ± SD (*n* = 3). Statistical analysis was performed using a linear model followed by Tukey-adjusted pairwise comparisons. *P < 0.05; **P < 0.01; ns, not significant.

bioRxiv preprint doi: https://doi.org/10.64898/2026.09.15.751907; this version posted September 17, 2026. The copyright holder for this preprint (which was not certified by peer review) is the author/funder, who has granted bioRxiv a license to display the preprint in perpetuity. It is made available under a CC-BY 4.0 International license.

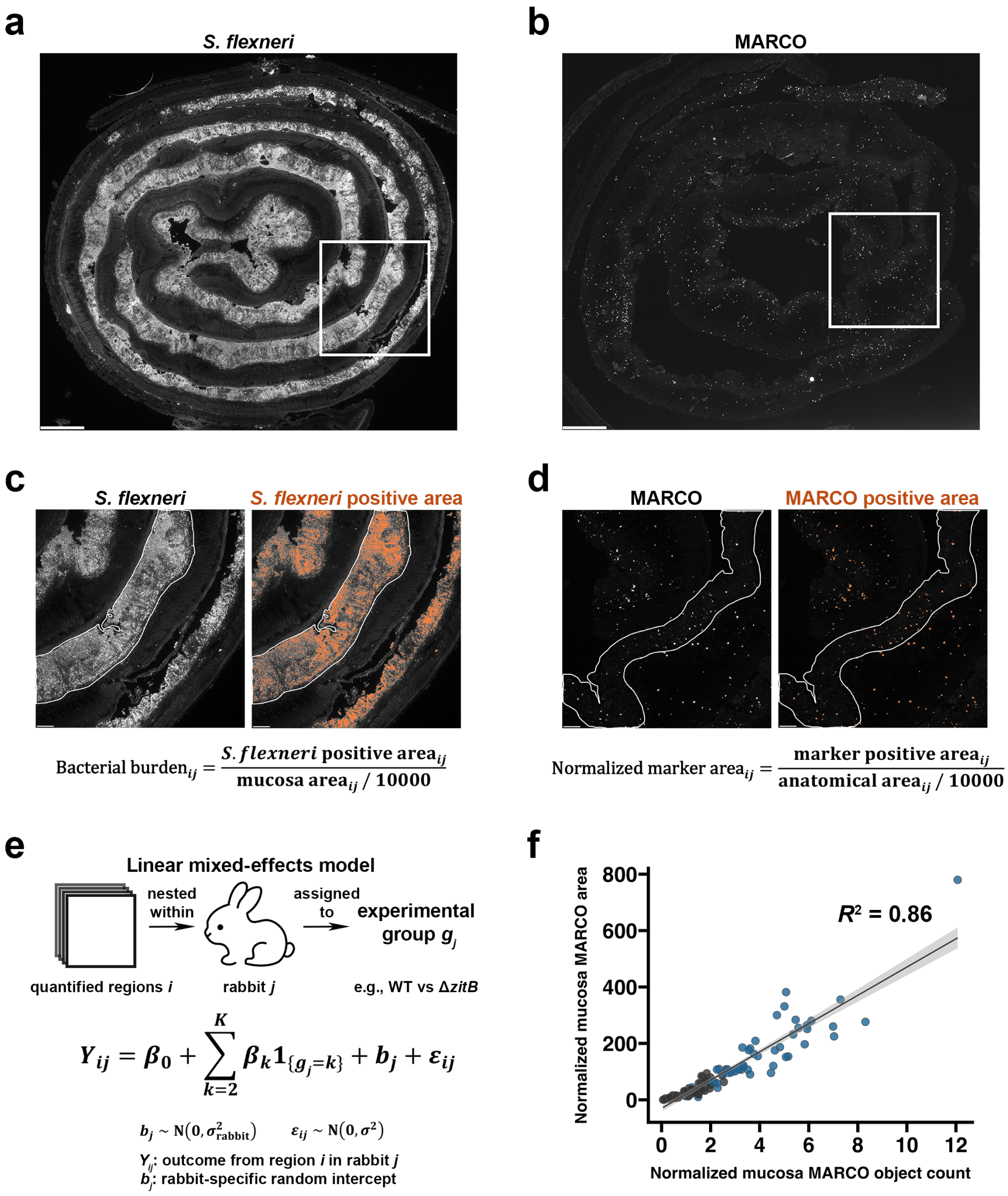

Supplementary Fig. 5: Workflow for tissue image quantification and mixed-effects modeling.

a–**b** Representative montages of colon Swiss rolls showing *S. flexneri* signal (**a**) and *MARCO* signal as an example of RNAscope marker imaging (**b**). Each montage was assembled from acquired images to provide an overview of the tissue section. White rectangles indicate the acquired images shown in **c** and **d**, respectively. Scale bars, 500 µm. **c**–**d** Acquired images indicated in **a** and **b**, respectively, showing the original *S. flexneri* (**c**) or *MARCO* (**d**) signal and the corresponding thresholded positive area in orange. White lines delineate the mucosa. Signal-positive area and the corresponding anatomical area were measured within each quantified region. Bacterial burden was normalized to mucosal area (**c**), whereas marker-positive area was normalized to the anatomical area analyzed (**d**). Normalized values were reported using a 100 px × 100 px reference area (10,000 px^2). The marker-quantification workflow was applied to *MARCO* and *LCN2* in the mucosa, submucosa, and muscle. Scale bars, 100 µm. **e** Schematic of the hierarchical analysis. Quantified regions were nested within rabbits, which were assigned to experimental groups, and outcomes were analyzed using linear mixed-effects models with experimental group as a fixed effect and rabbit as a random intercept. **f** Relationship between normalized mucosal *MARCO*-positive area and normalized mucosal *MARCO* object count in a validation subset of 113 quantified regions from rabbits infected with WT or Δ*zitB* rabbits (n = 4 rabbits per group). Each point represents one quantified region; gray and blue indicate WT and Δ*zitB*, respectively. The line shows the linear regression fitted to the combined data from both groups; shading indicates the 95% confidence interval (R^2 = 0.86).

bioRxiv preprint doi: https://doi.org/10.64898/2026.09.15.751907; this version posted September 17, 2026. The copyright holder for this preprint (which was not certified by peer review) is the author/funder, who has granted bioRxiv a license to display the preprint in perpetuity. It is made available under a CC-BY 4.0 International license.

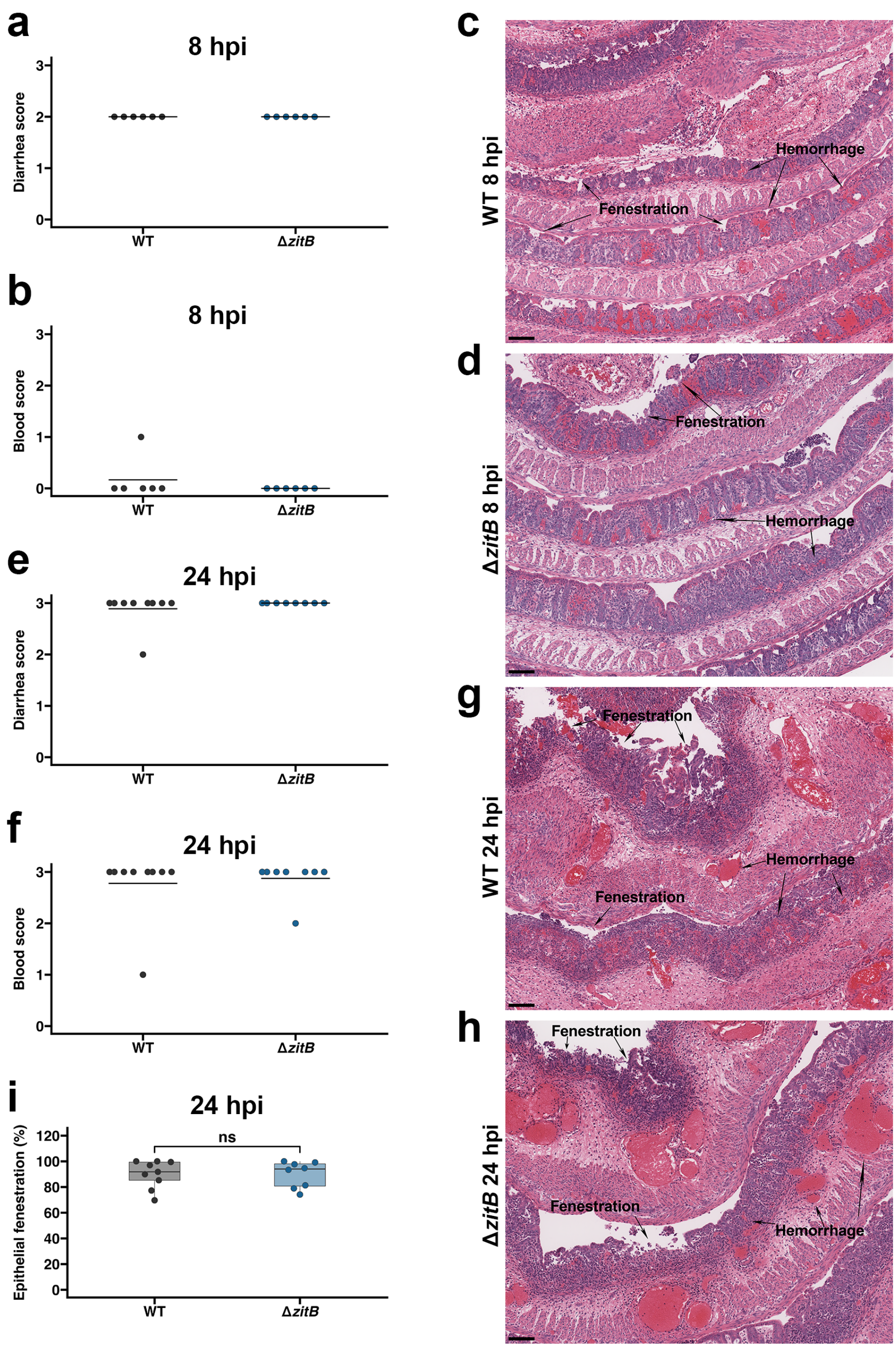

Supplementary Fig. 6: WT and Δ*zitB* infections show comparable intestinal pathology and epithelial fenestration.

a–**b** Diarrhea (**a**) and blood (**b**) scores in rabbits infected with WT or Δ*zitB* at 8 hpi. Each point represents one rabbit, and horizontal lines indicate the mean (*n* = 6 per group). **c**–**d** Representative hematoxylin and eosin (H&E)-stained colon sections from rabbits infected with WT (**c**) or Δ*zitB* (**d**) at 8 hpi. Arrows indicate examples of epithelial fenestration and hemorrhage. Scale bars, 100 µm. **e**–**f** Diarrhea (**e**) and blood (**f**) scores in rabbits infected with WT or Δ*zitB* at 24 hpi. Each point represents one rabbit, and horizontal lines indicate the mean (*n* = 9 for WT and *n* = 8 for Δ*zitB*). **g**–**h** Representative H&E-stained colon sections from rabbits infected with WT (**g**) or Δ*zitB* (**h**) at 24 hpi. Arrows indicate examples of epithelial fenestration and hemorrhage. Scale bars, 100 µm. **i** Percentage of the traced colonic epithelial length exhibiting fenestration at 24 hpi. Each point represents one rabbit (*n* = 9 for WT and *n* = 8 for Δ*zitB*). Boxes show the median and interquartile range (IQR); whiskers extend to the smallest and largest observations within 1.5 × IQR of the lower and upper quartiles, respectively. Statistical analysis was performed using a linear model. ns, not significant.

bioRxiv preprint doi: https://doi.org/10.64898/2026.09.15.751907; this version posted September 17, 2026. The copyright holder for this preprint (which was not certified by peer review) is the author/funder, who has granted bioRxiv a license to display the preprint in perpetuity. It is made available under a CC-BY 4.0 International license.

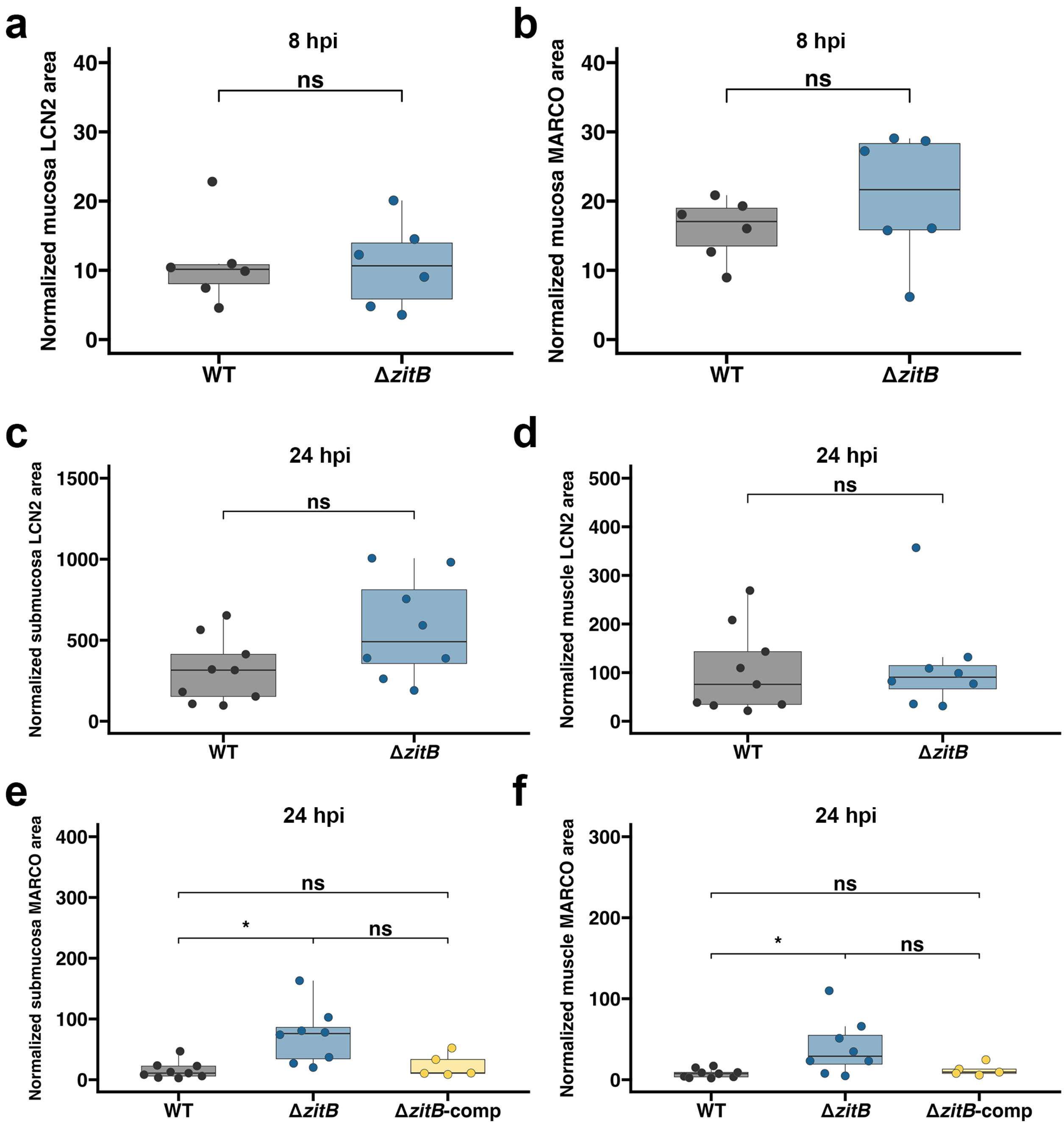

Supplementary Fig. 7: Additional analyses of *LCN2* and *MARCO* across intestinal tissue compartments.

a–**b** Normalized mucosal *LCN2*-positive area (**a**) and *MARCO*-positive area (**b**) in rabbits infected with WT or Δ*zitB* at 8 hpi (*n* = 6 per group). **c**–**d** Normalized submucosal (**c**) and muscle (**d**) *LCN2*-positive area in rabbits infected with WT or Δ*zitB* at 24 hpi (*n* = 9 for WT and *n* = 8 for Δ*zitB*). **e**–**f** Normalized submucosal (**e**) and muscle (**f**) *MARCO*-positive area in rabbits infected with WT, Δ*zitB*, or Δ*zitB*-comp at 24 hpi (*n* = 9 for WT, *n* = 8 for Δ*zitB*, and *n* = 5 for Δ*zitB*-comp). Each point represents the model-predicted mean for one rabbit, shown on the untransformed scale. Boxes show the median and IQR; whiskers extend to the smallest and largest observations within 1.5 × IQR of the lower and upper quartiles, respectively. Data were analyzed using linear mixed-effects models with strain as a fixed effect and rabbit as a random intercept. Models for **a**–**c** were fitted to untransformed values, whereas models for **d**–**f** were fitted to $\log_{10}$-transformed values; Pairwise comparisons in **e**–**f** were Tukey-adjusted. *$P < 0.05$; ns, not significant.

bioRxiv preprint doi: https://doi.org/10.64898/2026.09.15.751907; this version posted September 17, 2026. The copyright holder for this preprint (which was not certified by peer review) is the author/funder, who has granted bioRxiv a license to display the preprint in perpetuity. It is made available under a CC-BY 4.0 International license.

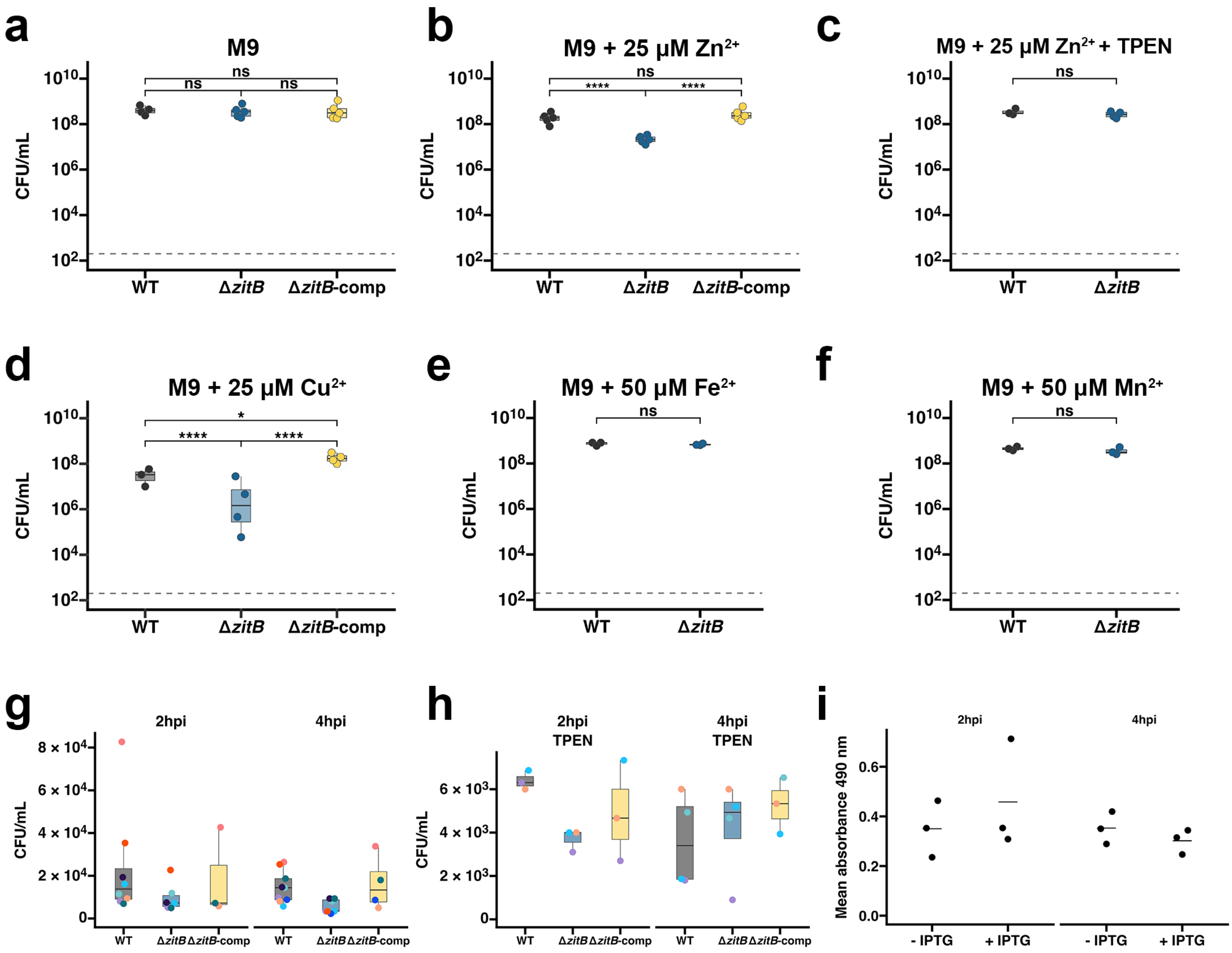

Supplementary Fig. 8: Metal-dependent growth and additional analyses of THP-1 macrophage infection.

a–**f** Endpoint CFU after overnight growth in M9 medium without added metal (**a**) or supplemented with 25 µM Zn^{2+} (**b**), 25 µM Zn^{2+} and 25 µM TPEN (**c**), 25 µM Cu^{2+} (**d**), 50 µM Fe^{2+} (**e**), or 50 µM Mn^{2+} (**f**). Each point represents the mean CFU from one independent experiment. Sample sizes were: **a**, n = 4, 5, and 5 for WT, Δ*zitB*, and Δ*zitB*-comp, respectively; **b**, n = 5 per group; **c**, n = 3 and 4 for WT and Δ*zitB*; **d**, n = 3, 4, and 4, for WT, Δ*zitB*, and Δ*zitB*-comp respectively; and **e**–**f**, n = 3 per group. Dashed lines indicate the limit of detection, 2×10^2 CFU ml^{-1}. Data were analyzed using linear models fitted to log_{10}-transformed values; pairwise comparisons in **a**, **b**, and **d** were Tukey-adjusted. **g**–**h** Raw CFU measurements from the THP-1 macrophage infection experiments shown in Fig. 5a–b, performed without TPEN (**g**) or with TPEN (**h**) at 2 and 4 hpi. Each point represents one independent infection experiment, and colors indicate experimental batch. In **g**, at 2 and 4 hpi, respectively, n = 8 and 9 for WT, n = 6 and 7 for Δ*zitB*, and n = 3 and 4 for Δ*zitB*-comp. In **h**, at 2 and 4 hpi, respectively, n = 3 and 4 for WT, 3 and 4 for Δ*zitB*, and 3 and 3 for Δ*zitB*-comp. For **a**–**h**, boxes show the median and IQR; whiskers extend to the smallest and largest observations within 1.5 × IQR of the lower and upper quartiles, respectively. **i** Mean absorbance at 490 nm for spontaneous LDH release controls consisting of uninfected THP-1 macrophages incubated with or without IPTG at 2 and 4 hpi. Each point represents one independent infection experiment (n = 3), and horizontal lines indicate the mean. *$P < 0.05$; ****$P < 0.0001$; ns, not significant.

bioRxiv preprint doi: https://doi.org/10.64898/2026.09.15.751907; this version posted September 17, 2026. The copyright holder for this preprint (which was not certified by peer review) is the author/funder, who has granted bioRxiv a license to display the preprint in perpetuity. It is made available under a CC-BY 4.0 International license.

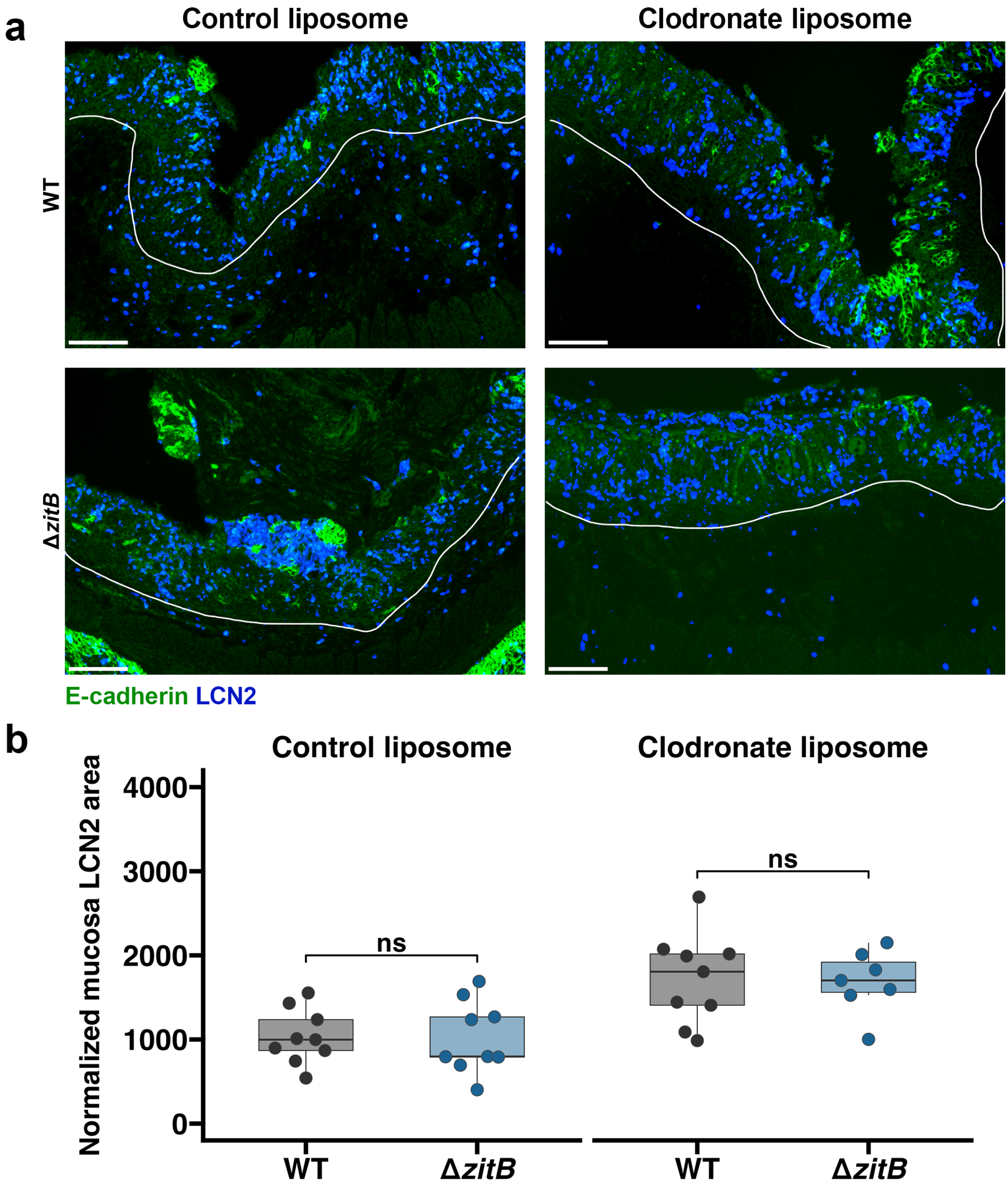

Supplementary Fig. 9: WT and Δ*zitB* infections show comparable mucosal *LCN2* signal following clodronate liposome treatment.

a Representative images of colonic tissue from rabbits treated intravenously with control or clodronate liposomes 48 h before infection with WT or Δ*zitB* and collected at 24 hpi. *LCN2* is shown in blue and E-cadherin in green. White lines delineate the boundary between the mucosa and submucosa. Scale bars, 100 μm. **b** Quantification of normalized mucosal *LCN2*-positive area following control or clodronate liposome treatment. Each dot represents the model-predicted mean for one rabbit. Sample sizes were n = 9 for WT and n = 8 for Δ*zitB* following control liposome treatment and n = 9 for WT and n = 7 for Δ*zitB* following clodronate liposome treatment. Data were analyzed using a linear mixed-effects model strain and treatment as fixed effects and rabbit as a random intercept. The same strain effect estimated by the additive model is indicated in both treatment panels. Boxes show the median and IQR; whiskers extend to the smallest and largest observations within 1.5 × IQR of the lower and upper quartiles, respectively. ns, not significant.
